# Extreme Phenotype Sampling and Next Generation Sequencing to Identify Genetic Variants Associated with Tacrolimus in African American Kidney Transplant Recipients

**DOI:** 10.21203/rs.3.rs-4050136/v1

**Published:** 2024-03-14

**Authors:** Moataz Mohamed, Bin Guo, Baolin Wu, David Schladt, Amutha Muthusamy, Weihua Guan, Juan Abrahante, Guillaume Onyeaghala, Abdelrahman Saqr, Nathan Pankratz, Gaurav Agarwal, Roslyn Mannon, Arthur Matas, William Oetting, Rory Remmel, Ajay Israni, Pamala Jacobson, Casey Dorr

**Affiliations:** University of Minnnesota; University of Minnesota; University of Minnesota; University of Minnesota; Department of Experimental and Clinical Pharmacology, College of Pharmacy, University of Minnesota; Hennepin Healthcare Research Institute

## Abstract

African American (AA) kidney transplant recipients (KTRs) have poor outcomes, which may in-part be due to tacrolimus (TAC) sub-optimal immunosuppression. We previously determined the common genetic regulators of TAC pharmacokinetics in AAs which were CYP3A5 *3, *6, and *7. To identify low-frequency variants that impact TAC pharmacokinetics, we used extreme phenotype sampling and compared individuals with extreme high (n=58) and low (n=60) TAC troughs (N=515 AA KTRs). Targeted next generation sequencing was conducted in these two groups. Median TAC troughs in the high group were 7.7 ng/ml compared with 6.3 ng/ml in the low group, despite lower daily doses of 5 versus 12mg, respectively. Of 34,542 identified variants across 99 genes, 1,406 variants were suggestively associated with TAC troughs in univariate models (p-value <0.05), however none were significant after multiple testing correction. We suggest future studies investigate additional sources of TAC pharmacokinetic variability such as drug-drug-gene interactions and pharmacomicrobiome.

## Introduction

Kidney transplantation is the treatment of choice for end-stage kidney disease [1,2]. African Americans (AAs) have the highest prevalence of end-stage kidney disease in the United States [3]. Compared to other ancestries, AA kidney transplant recipients (KTRs) have worse outcomes after kidney transplantation. AA KTRs have increased risk for acute rejection and delayed graft function [4]. Moreover, AA KTRs have lower graft and patient survival and the lowest five-year graft survival compared to the other ancestries [5,6].

Lifelong immunosuppressive therapy is required post-transplantation to prevent organ rejections [2]. Tacrolimus (TAC) is a calcineurin inhibitor immunosuppressant that is metabolized by the cytochrome P450 (CYP) 3A4 and CYP3A5 enzymes [7]. TAC is widely used in transplantation as a first line immunosuppressant and is used in ~90% of KTRs [2,8]. TAC has a narrow therapeutic index; underexposure is associated with graft rejection and loss while overexposure increases the risk of toxicity and opportunistic infections [9–11]. Additionally, TAC has high intra and inter-patient pharmacokinetic (PK) variability [12]. High variability of TAC troughs is associated with allograft loss, acute and chronic rejection [13,14]. Achieving optimal TAC troughs is challenging and mainly relies on a trial-and-error approach which generally requires multiple dose adjustments. We aim to develop pharmacogenomic-based TAC dosing to improve the efficacy and reduce intrapatient TAC trough variability that may improve outcomes.

Because the active form of *CYP3A5* (*1 allele) is most common in people of African ancestry, AAs are more likely to express active CYP3A5 enzyme leading to higher TAC clearance and dose requirements [15]. However, not all AAs have the *1 CYP3A5 allele. Previously, we found that AAs had the lowest dose-adjusted TAC troughs despite the higher TAC daily dose compared to Americans with European, Native and Asian ancestries [15]. During the first 90 days post kidney transplant, high TAC clearance and low troughs were risk factors for acute rejection [16].

It is well established that common genetic variants in *CYP3A5* influence TAC PK and clearance in individuals with African ancestry. We conducted a genome-wide association study (GWAS) in AA KTR and found that dose adjusted TAC troughs were highly associated with three loss-of-function (LoF) genetic variants in *CYP3A5* gene, CYP3A5*3 (rs776746), CYP3A5*6 (rs10264272) and CYP3A5*7 (rs41303343) [12]. Clinical factors and these genetic LoF variants in the *CYP3A5* gene explained 53.9% of the observed interpatient variability in troughs [12].Dosing equations using population PK studies are developed for TAC in AAs and account for these three LoF common variants [17,18].

We hypothesized that TAC trough interpatient variability is partially due to rare genetic variants in genes related to TAC disposition and could be identified through extreme phenotype sampling (EPS) and next generation sequencing (NGS). We previously developed a targeted EPS and NGS approach to identify novel genetic variants in 24 European American and 24 AA KTRs [19]. This study identified an association of *CYB5R2* with TAC troughs in AAs. Within *CYB5R2*, rs61733057 was predicted to disrupt protein function. Recently, Collins et al, [20] discovered novel genetic variants in the distal regulatory region (DRR) of *CYP3A* locus. These variants were reported to influence *CYP3A4* and *CYP3A5* expression and may impact drug metabolism in humans. Two of these variants were exclusively present in AAs (rs115025140 and rs111266634). Additionally, the rs776744 and rs776742 variants were reported to be associated with TAC troughs in Chinese KTRs [20].

Considering these findings, the aims of this study were to identify potential new variants, evaluate the *CYB5R2* variant (rs61733057) in a larger population, and study the association of the newly reported *CYP3A* DRR variants with TAC troughs in AA KTRs. In this new analysis we increased the NGS from 3.1 megabases in the previous study [19] to 5.3 megabases of genomic sequence. NGS was used because rare variants are likely absent in GWAS arrays used in earlier studies [12,15,21]. Identification of additional genetic variants that impact TAC troughs may improve understanding of TAC PK variability which ultimately may lead to improved treatment strategies for AA transplant recipients.

## Methods

### Study design and subjects selection

For this study, 128 KTRs were selected through EPS from self-identified AAs (n=515) enrolled in the observational multicenter prospective studies: Deterioration of Kidney Allograft Function (DeKAF) Genomics (clinicaltrials.gov, NCT00270712) and GEN03 genomics of kidney transplantation studies (clinicaltrials.gov, NCT01714440). Both studies were approved by Institutional Review Board at each transplant center. The DeKAF study sites were University of Alberta, University of Manitoba, University of Minnesota, Hennepin County Medical Center, Mayo Clinic, University of Iowa, and University of Alabama. The GEN03 sites included all the sites in DeKAF except the Canadian sites. Research participants were enrolled at the time of transplant after obtaining informed consent if they were ≥18 years old, received oral immediate release TAC as maintenance immunosuppression.

### Tacrolimus (TAC) trough measurements

TAC troughs and doses were obtained as part of routine clinical care for the first 6 months post-transplant. At each participating center, TAC troughs in whole blood were measured in Clinical Laboratory Improvement Amendments of 1988 certified laboratories by liquid chromatography-mass spectrometry. All TAC troughs were at steady state. Doses were adjusted based on each participating center target troughs. However, in general target troughs were 8–12 ng/mL and 6–10 ng/mL for the first 3 months and 3–6 months post-transplant, respectively. TAC troughs (ng/mL) were normalized by the corresponding dose (mg).

### Extreme phenotype sampling

Linear mixed-effects modeling (LMM) was used to identify from the 515 AA recipients those with the lowest (fast TAC metabolism) and highest (slow TAC metabolism) dose normalized TAC troughs. To ensure linearity, TAC troughs were transformed to natural log (ln). The LMM used the ln-transformed dose-normalized TAC troughs adjusting for the clinical factors and the LoF genotypes, CYP3A5*3 (rs776746), CYP3A5*6 (rs10264272) and CYP3A5*7 (rs41303343), (Supplementary material, Table 1) collected from GWAS. Detailed methodology for GWAS is explained in our previous publications [12,21]. Clinical factors adjusted for: time post-transplant, transplant recipient age, simultaneous pancreas and kidney transplant, and antibody induction type and time-varying covariates glomerular filtration rate and antiviral use. The resultant multivariable model was used to determine TAC trough residuals which were then used to identify the participants with the extreme phenotypes of dose normalized TAC troughs at the highest and lowest 12.5 percentiles. The LMM included a random intercept, random slopes for days after transplant, and post-transplant day 9. The selection of a simple spline at day 9 post-transplant was based on our previous analyses which found that dose-normalized troughs initially start low, rise quickly until day 9 after transplant and then plateau in the early weeks after transplant [12,19].

### Targeted next generation sequencing (NGS)

DNA, isolated from peripheral blood and collected in the original studies, from participants in these extreme groups was subject to NGS. Hybridization-based capture was performed with 1 μg of genomic DNA with NimbleGen SeqCap EZchoice kit (Roche, NimbleGen). Targeted sequencing spanning the entire length of 60 genes (Table 1) was performed and extended ~20,000 base pairs upstream and downstream of these genes. Thus, the extended sequencing length included 39 partial genes adjacent to the 60 genes for a total of 99 genes (Supplementary material, Table 2) spanning 5.3 mega base pairs. These 60 genes were selected because they were reported in the literature as associated, or suspected association with TAC pharmacokinetics or pharmacodynamics. A custom coverage probe design (Roche, NimbleGen) was used allowing up to 20 close matches in the genome that increased the coverage across all regions. Standard SeqCap EZ gDNA libraries were developed and hybridized with the custom EZ choice probes following standard protocols. The captured libraries were multiplexed and sequenced with Illumina NovaSeq paired end sequencing (2 × 150 bp).

### Bioinformatics analysis of NGS data

FASTQC was used to evaluate the quality of raw Illumina sequences. Sequenced reads were aligned to University of California Santa Cruz’s human reference genome (GRCH 37/hg 19) with a Burroughs-Wheeler Aligner. We targeted >20x depth for making variant calls. Genome Analysis Toolkit’s best practices pipeline was used to identify and call variants. Quality control was performed on the called variants and were excluded if they had more than 2 alleles (multiallelic), only a single allele was observed, missing genotypes in >8% (> 10 participants), and/or out of Hardy-Weinberg Equilibrium after multiple testing correction using false discovery rate (FDR) at 5%. The Ensembl Variant Effect Predictor (VEP) tool was used to annotate the significant variants to their respective genes and predict coding consequences [22].

### Statistical analysis

Descriptive statistics were used to summarize participants’ characteristics and demographics. The TAC high vs low dose normalized trough participants were defined in a binary fashion as cases and controls. Single variants sequence kernel association test (SKAT) analysis for binary traits [23] was used to calculate p-values for the association of individual genetic variants from NGS in univariate analysis with TAC trough grouping. The *CYP3A* DRR variants from original GWAS were further tested in the full AA cohort (n=515) for association with the dose adjusted TAC troughs using LMM while adjusting for the same covariates in the EPS model. Burden, SKAT and SKAT-O tests were used for gene-based association analyses with TAC groups. Suggestive association was defined by a p-value <0.05 before multiple testing correction. Significant association was defined after multiple testing correction by false discovery rate at <0.05. Analyses were conducted with SAS version 9.3 software (SAS Institute, Cary, NC) and R software (version 4.3.0, Vienna, Austria).

## Results

### Subject characteristics and demographics

**Figure 1:**
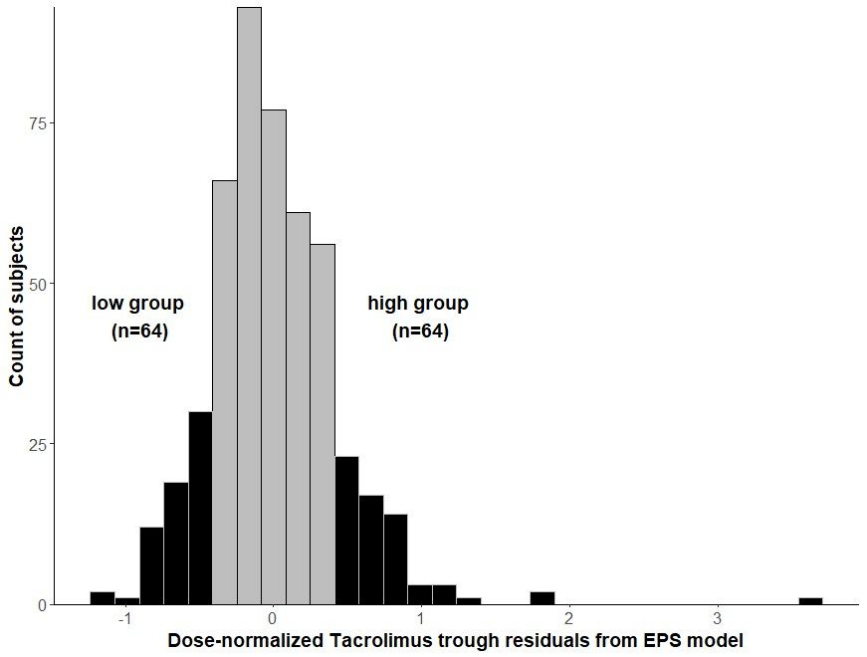
The distribution of the Extreme Phenotype Sampling (EPS) tacrolimus trough residuals from 128 African American (AA) kidney transplant recipients. The graphs represent the histogram of the residuals from the longitudinal linear mixed-effects model after adjusting for genetic variants CYP3A5 *3, *6, and *7 and clinical factors. Out of a total sample of 515 AA kidney transplant recipients, 128 research participants were selected using the linear mixed-effects model representing the 12.5% of the highest and 12.5% of the lowest dose-normalized tacrolimus troughs and then classified into high TAC group and low TAC group with 64 participants in each group.

Table 2 shows the demographics and characteristics of research participants. The EPS model identified 128 participants out of 515 with the lowest (low TAC) and highest (high TAC) dose-normalized TAC troughs (64 participants in each group, Figure 1). Ten samples failed FASTQC sequencing quality control; thus, the final EPS sample was 58 participants in the high TAC group and 60 participants in the low TAC group. Sixty-two % and 25% participants in the high and low TAC groups were females, respectively. As designed in the model, the distribution of *CYP3A5* *3, *6 and *7 variants were almost identical in both groups and the majority (83% and 75.9% in low and high groups, respectively) of research participants had at least 1 loss of function variant in *CYP3A5* gene.

**Figure 2:**
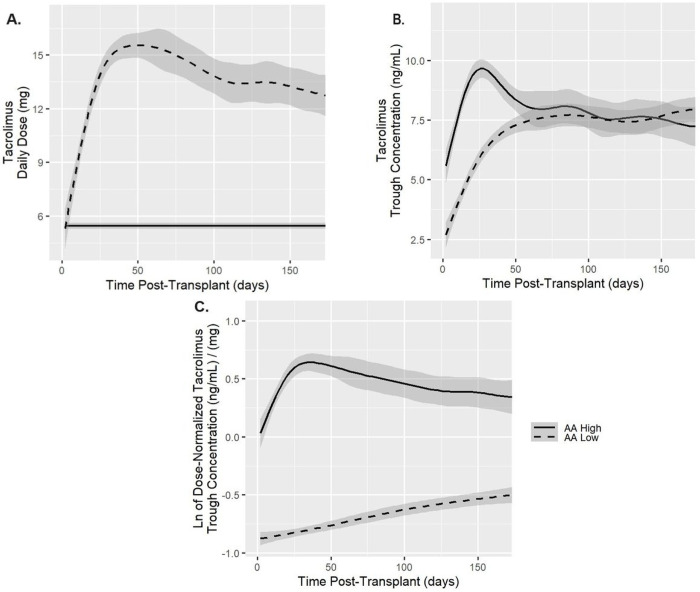
Tacrolimus troughs and doses in high and low groups. The lines represent the fitted trend line, and the gray band represents the 95% confidence interval. Plot A represents tacrolimus daily dose. The low group (dashed line) received a higher tacrolimus daily dose compared to the high group (solid line). Plot B represents dose-unnormalized tacrolimus troughs in the high and low groups. In both groups, the troughs started to plateau around day 60 post-transplantation. Plot C represents the natural log (ln) of the dose-normalized tacrolimus troughs. The troughs in the high group are higher than the low group and the troughs of the low group continuously increase with time post-transplant.

The high TAC group had a median TAC trough concentration of 7.7 ng/ml compared to the low TAC group with median of 6.3 ng/ml despite a higher median daily dose in the low TAC group of 12 mg vs only 5 mg for the high TAC group. Similarly, the median dose-normalized TAC troughs in the high TAC group were ~3 times higher than the low TAC group (median, 1.46 vs 0.48 ng/mL/mg, respectively). For the first 6 months post-transplant, the high TAC group had higher unnormalized and dose-normalized troughs while the low TAC group had a higher daily dose compared to the high TAC group. The low TAC group had a higher mean trough IPV compared to the high TAC group (43.77% ± 15.24% vs 39.29% ± 9.54%). The trends in TAC troughs post-transplant are presented in Figure 2.

### Identified variants

We identified 41,510 variants across the 60 fully sequenced and 33 partially sequenced genes from the GRCH 37/hg 19 reference genome. The majority (48%) of the identified variants were intronic while 24% were intergenic, 1.3% were exonic and 26.7% were defined as other. A final number of 34,542 genetic variants were maintained after quality control and tested for association with TAC troughs (Supplement material, Figure 1).

### Associations of individual variants with TAC trough high and low groups

Using the SKAT binary test, we evaluated the identified 34,542 variants for association with EPS TAC groups (high TAC vs low TAC). None of these variants were significantly associated with either TAC groups after adjusting for the multiple testing using a FDR threshold of 0.05. However, 1,666 variants were suggestively associated with EPS TAC groups (high TAC vs low TAC) (p-value<0.05 before FDR). Detailed p-values for the 34,542 genetic variants are reported in Supplementary material, Table 3. For each gene, the number of suggestive variants that were associated with TAC EPS high and low groups was calculated, top 25 genes are presented in Table 3. *PPP3CA, ABCC4* and *SLCO1B1* were the top 3 genes with 236, 85 and 82 variants suggestively associated with TAC troughs, respectively. The full list of the number of suggestive variants in each gene is listed in Supplementary material, Table 4.

### VEP analysis for suggestive variants

Among the 1,666 variants suggestively associated with TAC troughs, 1,406 were successfully processed by VEP tools (Supplementary material, Table 5). VEP identified 16 (1.1%) variants as novel. Most of these variants were intronic (58%) and synonymous variants (58%) while 42% were missense variants. The summary of the VEP analysis is presented in Supplement material, Figure 2.

### *CYP3A4* and *CYP3A5* variants

Ten variants suggestively associated with TAC troughs were identified in the CYP3A5 (7 variants) and CYP3A4 (3 variants) genes. Only one (CYP3A4*15, rs4986907, chr7:99367427) of these 10 variants was a missense variant therefore having biologic rationale for potential impact on CYP3A4 function. Most of the observed carriers of the CYP3A4*15 variant were in the high TAC group (Table 4) further suggesting that CYP3A4*15 is an important LoF variant.

### *CYP3A* distal regulatory variants

Genetic variants rs776744 and rs776742 were found to be in complete linkage disequilibrium (D’= 0.999, R^2^ = 0.999), therefore only rs776744 was tested for an association with TAC troughs. The CYP3A distal regulatory variants (rs776744, rs115025140 and rs111266634) were not associated with TAC troughs in the full (n=515) nor EPS (n=118) AA cohorts, Table 5.

### Gene-based association analyses

Gene based association test was performed for the 99 fully and partially sequenced genes using burden, SKAT and SKAT-O tests. Only 9 genes were associated with TAC groups (high vs low) at p-value <0.05, Table 6. However, none of these genes maintained their statistical significance after adjusting for multiple testing.

## Discussion

AAs KTRs have higher rates of graft loss compared to other populations, in part related to higher risk of developing acute rejection and this could be explained by the variability in immunosuppressive therapy due to high variability in TAC PK. AAs receive the highest dose of TAC compared to other populations, yet they have the lowest TAC troughs [15]. Approximately half of the variability observed in TAC trough concentrations in AAs is not explained by the three common CYP3A5 genetic variants and clinical factors [12]. Therefore, there is a need to identify the additional underlying sources of this unexplained variability to improve immunosuppressive outcomes. We conducted this EPS study to identify genetic variants that could be associated with TAC troughs in AAs.

EPS is an efficient design to identify rare variants without the need of large-scale studies [24,25]. An advantage of using EPS is the ability to account for potential covariates in the model [19]. Moreover, EPS can identify participants with extreme phenotypes and may limiting the need of conducting NGS over the whole cohorts to save time and cost. To overcome some of the limitations of our previous study [19], including a small sample size, targeting only 28 full genes, and not accounting for multiple testing, we designed the current study with a larger cohort of AA and with a larger number of targeted genes and more rigorous testing.

The CYP3A4*15 (rs4986907, minor allele frequency in AA =0.0257) is a rare missense genetic variant that is almost exclusively found in populations of African ancestry [26]. In the current study, the CYP3A4*15 was more frequent in the high group (slow metabolizers) suggesting that it creates an enzyme with absent or decreased function. In vitro, the CYP3A4*15 variant was reported to have a decreased relative clearance (60%) of oxycodone compared to CYP3A4*1 [27]. Midazolam metabolism was slower in the *CYP3A4* *15 as we would expect in a missense CYP3A4 variant enzyme, which may be consistent with our data in this study [28]. TAC and midazolam might have similar metabolic mechanisms as shown in our CRISPR based drug metabolism assay model [29]. However, in a large series of in vitro studies, the kinetics for a variety of substrates (not including TAC) were determined for the wild-type enzyme, CYP3A4*1 versus >20 *CYP3A4* variants including CYP3A4*15. For most of the studied substrates the intrinsic clearance was higher in CYP3A4*15. The enzymatic kinetics parameters of CYP3A4*1 and CYP3A4*15 and relevant references are presented in supplementary material, Table 6. The evidence for the enzymatic function of this variant is conflicting. Therefore, it should be tested with TAC in cell culture potentially by using the CRISPR engineered cell lines to define the impact on TAC metabolism [29].

Collins and colleagues [20] reported variants in the CYP3A DRR that increased expression of *CYP3A4* and *CYP3A5* mRNA and/or protein. The rs115025140 variant also showed a trend towards reduced statin efficacy. While rs111266634 variant showed a reduced reporter expression in luciferase reporter assay without affecting *CYP3A4* and *CYP3A5* gene expression [20]. This study also reported that the rs776744 and rs776742 variants, which are not exclusive in AAs, were associated with TAC trough concentrations in Chinese KTRs. Because of the potential influence of these variants on CYP3A4 and CYPA5 gene and/or protein expression, we assessed their association with TAC in this study and found that none of these regulatory variants were associated with TAC troughs. Although these variants may influence the expression of CYP3A4 and CYP3A5in vitro, their clinical impact may be modest as their effects might be masked by the influence of the known genetic variants in *CYP3A4* and *CYP3A5*.

Between our previous GWAS studies in DeKAF and GEN03, and our two EPS and NGS studies, we and others have likely identified the most important common variants associated with TAC metabolism in AAs kidney transplant recipients, CYP3A5 *1, *3, *6, *7 [21]. There may be other important genetic variants associated with TAC, but since they were not identified in these genetic studies and if present, are likely in very low frequency.

We hypothesize that drug-drug-gene interactions may obscure the genetic influence of a single variant. KTRs take on average 10–15 drugs per day [30–32] and some of these drugs may phenoconvert the genetically predicted CYP3A4 and CYP3A5 phenotypes to an alternative phenotype by introducing a drug interaction that confounds the interpretation of the variant function [33,34]. Drug-drug-gene interactions with TAC should be investigated in patients and in cell culture studies that eliminate the environmental and social factors which also play a role in TAC trough variability. Moreover, we can investigate the influence of low frequency variants, and drug-drug-gene interactions on TAC metabolism in cell culture methods [29]. Use of modeling tools that predict in vivo drug-drug-gene interactions using in vitro data could hasten the identification of these interactions [35–37].

There are other factors that could potentially impact TAC PK variability such as microbiome and epigenetics. There is growing evidence that the gut microbiome could have a potential association with TAC PK variability. In vitro, TAC was found to be metabolized by some gut microbiome to a bacterial specific and less active C-9 keto reduction TAC metabolite [38,39]. Moreover, TAC dose and/or trough variability was found to be associated with gut microbiome in solid organ transplants and the C-9 keto reduction metabolite was found in the blood of transplant patients [40].

The current study has limitations. First, we used targeted NGS which is considered limited compared to whole genome or whole exome sequencing. To overcome this limitation, we targeted most of the genes that have been linked to TAC pharmacokinetics and/or pharmacodynamics in the literature. Although in the current study we had a larger sample size compared to our previous EPS study [19], the sample size is still small. This could affect the power of the study as most of the suggestive variants are infrequent and will require more participants to conclude that these variants are important. In the current study, we did not account for TAC adherence although two recent study results showed TAC adherence did not significantly affect the TAC trough variability in KTRs [41,42]. Although we collected information on concomitant medications, we only adjusted the analyses for broad drug classes. Agents of the same drug class do not have the same magnitude of drug-drug and/or drug-drug gene interactions. Therefore, in future studies, it would be important to account for the use of each agent separately and not as a whole class. We do not have detailed information on all concomitant CYP3A4 and/or CYP3A5 inhibitors or inducers, and this is important in future studies.

## Conclusion

There were broad differences in dosing and TAC troughs that we identified in this study between the high and low groups in AA KTRs. None of the identified genetic variants were significantly associated with TAC troughs using the EPS model after accounting for the CYP3A5*3, *6, *7 variants. However, some genetic variants were found to be suggestively associated with TAC troughs and require additional evaluation. Since there are large differences in TAC PK within this group of KTRs, future studies should investigate other sources of variability such drug-drug gene interactions, epigenetics and microbiome as contributors to the unexplained TAC PK variability.

## Data Availability

*Pending Acceptance* - Raw sequence data of this study, in FASTQ format, will be available at the United States National Center for Biotechnology Information’s (NCBI) Sequence Read Archive (SRA) and the associated phenotype and covariate data will be available at NCBI’s Database for Genotypes and Phenotypes with dbGaP.
